# Moral judgement under induced anxiety: threat-of-shock reduces sensitivity to immoral acts and alters neural processing

**DOI:** 10.1093/scan/nsaf093

**Published:** 2025-09-10

**Authors:** Jiaping Cheng, Jianhui Wu, Fang Cui

**Affiliations:** School of Psychology, Shenzhen University, Shenzhen, 518061, China; School of Psychology, Shenzhen University, Shenzhen, 518061, China; Center for Brain Disorders and Cognitive Neuroscience, Shenzhen University, Shenzhen, 518061, China; School of Psychology, Shenzhen University, Shenzhen, 518061, China; Center for Brain Disorders and Cognitive Neuroscience, Shenzhen University, Shenzhen, 518061, China

## Abstract

This study investigates how anxiety influences moral judgement processes using event-related potential (ERP) techniques. Participants were instructed to rate their feelings towards others’ moral and immoral acts while neural responses were recorded under safe and threat-of-shock (TOS) conditions. Participants reported significantly higher anxiety levels in the TOS context, accompanied by increased non-specific skin conductance responses (NSSCR), indicating heightened autonomic nervous system activity. Behaviourally, participants in the TOS context rated immoral behaviours as significantly less unpleasant compared to those in the safe context, while ratings for moral behaviours did not differ significantly, suggesting reduced sensitivity to immoral acts in the TOS context. ERP results revealed larger N1 amplitudes in response to immoral behaviours in the TOS condition, reflecting heightened attention to threatening stimuli. In contrast, the N400 component showed significant differences between moral and immoral acts only in the safe condition; this distinction was absent in the TOS condition, indicating impaired semantic processing under anxiety. Together, these findings demonstrate that threat-induced anxiety disrupts moral judgement processes, leading to reduced sensitivity to immoral behaviours. This highlights the critical role of anxiety in moral processing and the flexibility and context dependence of moral judgements.

## Introduction

Moral judgement—the evaluation of the moral rightness or wrongness of actions (Malle 2021, Skitka et al 2021)—is fundamental for establishing social norms, facilitating interactions, and fostering societal bonds. Such judgement emerges from an interplay between emotionally driven intuitive responses and rational cognitive integration, each characterized by distinct temporal dynamics (Greene 2003, Greene et al 2004, Greene 2014).

Emotional states, particularly anxiety, significantly shape moral judgement (Starcke et al 2012, Youssef et al 2012, Kouchaki and Desai 2015, Singer et al 2021). According to the emotion amplification hypothesis, negative emotions like anxiety intensify intuitive moral reactions (Haidt 2001, Horberg et al 2011). Under heightened anxiety, individuals tend to make harm-avoidance judgements, relying more on intuition than deliberate reasoning (Gan et al 2015, Tinghog et al 2016, Białek and De Neys 2017, Bago and De Neys 2019, Brown et al 2020). Threat contexts robustly amplify negativity bias across emotional and perceptual domains (Baker et al 2020, Norris 2021), increase risk perception (McEwen 2012, Phelps et al 2014), and promote reliance on social norms for psychological safety (Ellemers et al 2002, Tyler and Blader 2003). Neurologically, moral evaluation engages the amygdala and ventromedial prefrontal cortex (vmPFC) (Li et al 2021); anxiety enhances amygdala activity (van Marle et al 2009, Shackman et al 2011) while impairing prefrontal function (Shin and Liberzon 2010, Arnsten et al 2015). This complex interplay underscores the need for further investigation into anxiety’s role in moral cognition.

Previous research on anxiety’s impact on moral judgement has predominantly focused on harm-related behaviours (Katrin et al 2011, Zhao et al 2016, Li et al 2021), often neglecting broader moral violations such as violations of social norms. Addressing this gap is essential for a comprehensive understanding of anxiety’s influence on moral processing. Event-related potentials (ERPs), with their high temporal resolution, offer a powerful tool to capture the neural dynamics underlying moral processing. Accordingly, this study aims to elucidate the temporal course of moral judgement under induced anxiety using ERP measures.

Recent studies have highlighted how anxiety disorders disrupt prefrontal-limbic circuits critical for moral judgement. For example, Patil et al (2021) found that individuals with generalized anxiety disorder show heightened harm aversion in moral dilemmas, associated with hyperactivity in the anterior insula and reduced engagement of the vmPFC. Complementing this, Zhang (2025) meta-analysis demonstrated that acute stress shifts moral processing towards amygdala-driven intuitive responses, impairing prefrontal regions involved in deliberative reasoning. Building on these findings, we hypothesize that threat-induced anxiety in our paradigm biases moral judgement towards rapid, affectively driven processing—reflected in modulation of early ERP components—while diminishing controlled, integrative processes indexed by later components.

We employed a threat-of-shock (TOS) paradigm to induce anxiety while participants read sentences describing morally salient behaviours. Electroshock-induced anxiety is a validated approach eliciting immediate emotional responses (Schmitz and Grillon 2012, Robinson et al 2013, Weymar et al 2013, Balderston et al 2017, Bolton and Robinson 2017, Raio et al 2017, Jeong and Cho 2019, Chaisson 2021). To verify anxiety induction, we recorded electrodermal activity (EDA), with skin conductance responses (SCRs) serving as indicators of sympathetic arousal (Benedek and Kaernbach 2010).

We hypothesized that participants under the TOS context would exhibit elevated anxiety level and increased SCRs. Regarding moral judgements, two outcomes were considered: anxiety might heighten sensitivity to immoral behaviours by amplifying their threat salience, consistent with the emotion amplification hypothesis (Haidt 2001, Horberg et al 2011). Alternatively, anxiety might impair semantic processing due to cognitive overload, reducing sensitivity to moral violations and favouring heuristic judgements (Greene et al 2008, Paxton et al 2012). This disruption could lead individuals to rely more on automatic, heuristic judgements, therefore decrease the sensitivity to moral violations (Białek and De Neys 2017, Bago and De Neys 2019).

In the present study, we focused on three ERP components reflecting different stages of processing: early components N1 and P200, and later components N400 and P600. The N1 reflects early automatic attentional orienting and is typically enhanced under threatening (Hamilton et al 2014, Yang et al 2020). The N400 indexes cognitive effort in retrieving semantic and social knowledge, sensitive to moral and social incongruities (Chwilla et al 1995, De Pascalis et al 2009). Larger N400 amplitudes indicate increased processing when expectations are violated, making it a marker of moral evaluation (Bartholow et al 2001, White et al 2009). The P600 is linked to higher-order cognitive control, conflict monitoring, and re-evaluation during moral judgement, often elicited by morally complex or conflicting dilemmas (Sarlo et al 2012, Yoder and Decety 2014). Collectively, these components provide a temporal map of how anxiety modulates moral cognition from early attentional engagement to later deliberative processing.

## Materials and methods

### Participants

Thirty-seven right-handed individuals were recruited from Shenzhen University, all with normal or corrected-to-normal vision. Participants scoring more than two standard deviations above the mean on the Trait Anxiety Inventory (T-AI) or the Self-Rating Depression Scale were excluded during pre-screening (Shek 1988, Cheung 1996). One participant was further excluded due to excessive EEG artefacts, resulting in fewer than 75% of trials available for analysis. The final sample comprised 36 participants [16 males; mean age = 20.14 ± 2.22 years (mean ± standard deviation)]. All procedures complied with the Declaration of Helsinki (1964) and subsequent amendments, as well as relevant ethical standards. The study protocol was approved by the local ethics committee (protocol number: PN-202200067).

### Materials

**Figure 1.**
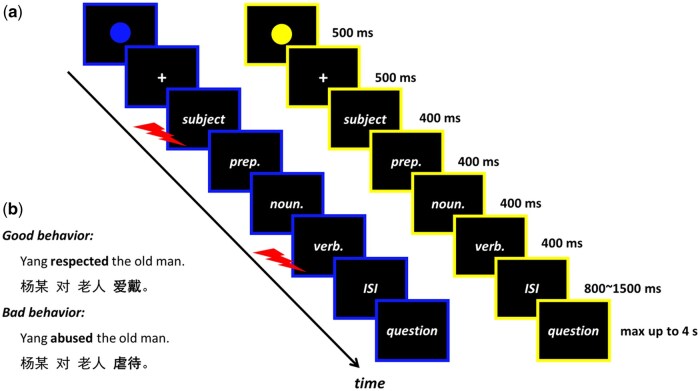
**Experimental design and materials**. (a) Schematic representation of example trials: the blue frame indicates threat-of-shock trials, while the yellow frame represents safe trials. The meanings of these colours were counterbalanced across participants. (b) Example sentences illustrating moral and immoral behaviours.

During the experiment, participants were presented with written sentences that described moral or immoral behaviours committed by specific agents. Each sentence consisted of four segments [i.e. subject + preposition + noun + verb (critical word)]. All sentences followed the same Chinese grammatical structure, with the final verb (i.e. the critical word) indicating the valence of the behaviour, as either moral or immoral. The moral and immoral behaviour materials were paired such that they depicted the same context but used opposite verbs to describe the actions performed by an agent. A total of 60 moral and 60 immoral behaviours were used. Eight surnames were selected from the ancient Chinese surname book *Hundred Family Surnames* to identify the agent described in the sentences (see Fig. 1a for an example). These materials were validated and used in previous studies (Xiaozhe et al 2017, Lu et al 2019).

The stimulus materials were pre-assessed by 31 independent subjects (16 females; mean age 22.84 ± 5.15 years). All behaviours showed significant moral relevance above baseline, confirming their moral attributes. Moral judgement scores significantly differed between moral and immoral behaviours. Consistent with Mende-Siedlecki et al (2013), immoral behaviours exhibited greater diagnostic weight. However, our primary hypotheses focus on interaction effects, ensuring that this baseline asymmetry does not impact our main findings. To provide the design of the stimulus materials clearly and visually, we have compiled all stimulus materials into a detailed table (see Supplementary Appendix Table 1).

### Experimental design and procedures

This study employed a 2 [Context: Threat of Shock (TOS) vs. Safe] × 2 (Behaviour of the Agent: Moral vs. Immoral) within-subject design. The formal experiment included four conditions: moral behaviour perceived under the TOS context *(TOS_Moral*), immoral behaviour perceived under the TOS context (*TOS_Immoral*), moral behaviour perceived under the Safe context (*Safe_Moral*), and immoral behaviour perceived under the Safe context (*Safe_Immoral*). Each condition consisted of 60 trials, resulting in a total of 240 trials. All trials were evenly divided into eight blocks, with participants given a 2–3-min rest after each block. The entire task lasted ∼25 min. To counterbalance the order of conditions, half of the participants completed the TOS blocks first, while the other half began with the Safe blocks.

Prior to the main experiment, a threshold measurement procedure was conducted to determine a painful yet tolerable shock intensity for each participant. This procedure began at a minimum intensity of 0.3 mA and increased incrementally by 0.5 mA, up to a maximum of 5.3 mA. Electrical stimulation was delivered using a constant stimulator (model: SXC-4A, Sanxia Technique, China) through two electrodes placed on participants’ non-dominant hands. Participants rated each shock on a scale from 1 (barely felt anything) to 9 (unbearable pain), with the threshold corresponding to a rating of 8 selected for use in the experiment. During the TOS blocks, participants were informed that they might receive up to four painful electrical shocks. In contrast, during the Safe blocks, participants performed the task without the threat of shocks.

Participants were seated ∼70 cm from the monitor, with a viewing angle of 2.1° × 0.6°. Each segment was presented in white font against a black background. At the beginning of each trial, a visual cue (a blue or yellow circle) was displayed for 500 ms to indicate whether the trial was under the TOS or Safe context (i.e. whether painful electrical shocks might be delivered during the task). A rectangle frame of the same colour was displayed throughout the trial to emphasize the condition, with the meanings of the colours counterbalanced across participants. Following a 500 ms fixation, each segment was presented for 400 ms, followed by a 400 ms blank screen. A random interval of 800 – 1500 ms was maintained between trials. In 48 randomly selected trials (12 trials per condition), participants rated their feelings about the behaviour on a nine-point Likert scale using buttons 1 to 9 on the keyboard, ranging from ‘1’ (extremely unpleasant) to ‘9’ (extremely pleasant). Participants were instructed to carefully watch the sentences presented on the screen and respond to any questions within 4 s (see Fig. 1b).

To ensure the effectiveness of anxiety induction, participants rated their subjective anxiety feelings on a nine-point Likert scale from 1 (not anxious at all) to 9 (extremely anxious) four times during the task. The stimulus display and behavioural data acquisition were conducted using E-Prime 3.0 professional software (Psychology Software Tools).

### Electrodermal activity measure

EDA was recorded to index sympathetic arousal via SCRs, reflecting autonomic reactions to stimuli(Benedek and Kaernbach 2010). Tonic nonspecific SCRs, which are not stimulus-locked, were also analysed (Boucsein et al 2012). Raw skin conductance data were acquired at 1000 Hz using the Biopac system, with two isotonic Ag/AgCl gel electrodes placed on the index and middle fingers of the participant’s dominant hand after cleaning the skin with 75% alcohol.

A 5-min resting baseline was recorded prior to the experiment, and data were subsequently extracted for each experimental block. Preprocessing and artefact correction followed established guidelines (Braithwaite et al (2013) using Biopac AcqKnowledge^®^ 5.0 software. To optimize processing, the sampling rate was downsampled to 15.625 Hz, preserving relevant physiological frequencies while reducing computational load. Slow drifts and tonic components were removed through smoothing and detrending, employing a 5-s baseline estimation window.

Phasic SCR peaks were identified within a 5-s window following stimulus onset, with all responses baseline-corrected by subtracting pre-stimulus values. Responses were classified as significant if their amplitude exceeded 0.02 μS and 10% of the session’s maximum SCR amplitude. Trials contaminated by movement artefacts or excessive noise were excluded from analysis.

### EEG data acquisition

Using a NeuroScan system, we continuously recorded EEG signals from 64 electrodes at 1000 Hz with the left mastoid as the online reference; ensuring scalp impedance was below 5 kΩ. The signals were bandpass filtered online from 0.1 to 100 Hz (AC). For offline analysis, we used the EEGLAB toolbox (Delorme and Makeig 2004) in Matlab to re-reference the data to the average of both mastoids and adjust the filter to 0.1 to 30 Hz (cutoff at −6 dB). We extracted segments from 200 ms pre-stimulus to 1200 ms post-stimulus, excluding shock trials. Baseline correction was performed using the mean voltage from the −200 to 0 ms pre-stimulus interval. Trials exhibiting excessive noise or movement artefacts were visually inspected and rejected by an experienced EEG analyst following established best practices. Independent Component Analysis (Makeig et al 2004, Onton et al 2005) was subsequently applied to identify and remove components associated with ocular, muscular, and other non-neural artefacts. Components reflecting eye blinks and movements were identified based on their temporal dynamics, scalp topography, and spectral characteristics and were removed accordingly. Following artefact correction, epochs containing residual amplitudes exceeding ±100 μV at any channel were automatically excluded from further analysis in accordance with committee guidelines.

### Data analysis

Time windows for each ERP component were determined based on the inspection of averaged data across all conditions and prior knowledge (Luck and Gaspelin 2017). The mean amplitude for each component was calculated as the arithmetic average of the selected electrode sites, based on the waveform and the scalp topographic map.

We focused on four key electroencephalographic (EEG) components that indicate distinct cognitive processes. The N1 component, associated with early attentional allocation and task-related stimulus detection, typically manifests in the prefrontal scalp region (Hillyard and Anllo-Vento 1998) with electrodes FCz, Cz, FC1, and FC2 selected for analysis (120–140 ms). The P2 component, reflecting early categorization and attentional processing, is usually observed in the prefrontal and central regions (Luck and Hillyard 1994, Clark and Hillyard 1996), and we analysed electrodes Fz, F1, F2, FCz, FC1, and FC2 (210–240 ms). The N400 component, linked to semantic activation and language processing, is typically distributed in the central-parietal region (Berkum et al 1999, Luo et al 1999, Lau et al 2008, Molinaro et al 2010), with electrodes C1, C2, Cz, CP1, CP2, and CPz chosen for analysis (320–370 ms). The P600 component, associated with syntactic processing and integration, is commonly found in the parietal midline region (Regel et al 2014, Ding et al 2016, Emerson et al 2020), using electrodes Pz, P1, P2, POz, PO3, and PO4 (500–800 ms).

We conducted a 2 (Context: TOS vs. Safe) × 2 (Behaviour: Moral vs. Immoral) repeated-measures analysis of variance (ANOVA) for each EEG component. Data analysis was performed using SPSS 26.0, with degrees of freedom and *P*-values adjusted using the Greenhouse–Geisser correction method. Descriptive statistics are reported as mean ± standard error (SE). This statistical approach enabled a detailed examination of the interactions between context and behaviour on cognitive processing, as reflected by the EEG components.

To investigate whether EDA moderated the relationship between experimental conditions and neural responses, we performed an exploratory moderation analysis using the PROCESS macro in SPSS (Hayes 2012). In this analysis, the independent variable (X) was the context (Safe/TOS), the dependent variable (Y) was the difference in neural responses, and the moderator (Mo) was EDA.

Although we balanced the order in which the safe and TOS conditions were implemented within subjects, we did some extended analyses to include condition *Order* as a covariate in the statistical analyses to observe the effect of the order variable on the overall results (see in Appendix S2).

## Results

### Validation of anxiety induction

**Figure 2.**
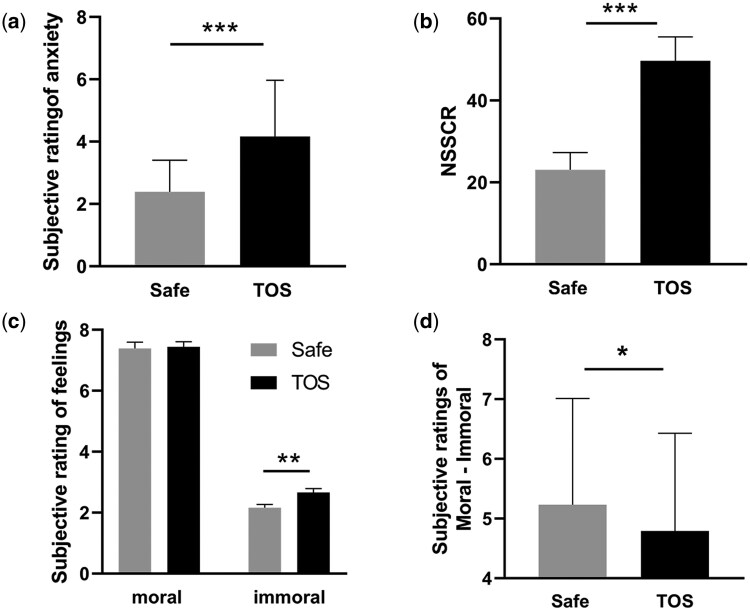
**Behaviour results**. (a) Self-reported anxiety levels in the TOS and Safe contexts; (b) Number of nonspecific skin conductance responses (NSSCRs) recorded in the TOS and Safe contexts; (c) Subjective ratings of feelings towards moral and immoral behaviours in the TOS and Safe contexts (1: extremely unpleasant, 5: neutral; 9: extremely pleasant); (d) Differences in subjective ratings between moral and immoral behaviours across the two contexts. Error bars represent the standard error. Notes: *p < 0.05, **p < 0.005, ***p < 0.001 (Bonferroni corrected).

To assess whether the threat of shock effectively induced anxiety, two-tailed paired *t*-tests were performed on participants’ self-reported anxiety levels between the Safe and TOS contexts. Results showed that anxiety ratings in the TOS context were significantly higher than in the Safe context (4.16 ± 1.81 vs. 2.39 ± 1.01, *t*_(35)_ = −6.67, *P* < .001) (Fig. 2a). For skin conductance data, two-tailed *t*-tests were used to analyse NSSCRs recorded in each context. NSSCRs in the TOS context were significantly greater than in the Safe context (49.67 ± 35.22 vs. 23.05 ± 25.39, *t*_(35)_ = −6.56, *P* < .001( Fig. 2b).

### Behavioural results

Subjective ratings of the agent’s behaviours across the four conditions were analysed using a 2 × 2 repeated-measures ANOVA. The main effect of context (*F*  _(1,35)_ = 10.346, *P* < .01, *η_p_*^2^ = 0.23) and behaviour (*F*  _(1,35)_ = 356.11, *P* < .01, *η_p_*^2^ = 0.91) were significant. Additionally, a significant interaction between Context and Behaviour was observed (*F*  _(1,35)_ = 4.48, *P* = .04, *η_p_*^2^ = 0.11). Pairwise comparisons revealed that for immoral behaviour, participants reported feeling less unpleasant in the TOS context compared to the Safe context (2.66 ± 0.13 vs. 2.16 ± 0.11, *P* < .001). However, for moral behaviour, no significant difference was observed between the two contexts (7.44 ± 0.17 vs. 7.39 ± 0.21, *P* = .74; Fig. 2c and d).

### ERP results

#### N1

**Figure 3.**
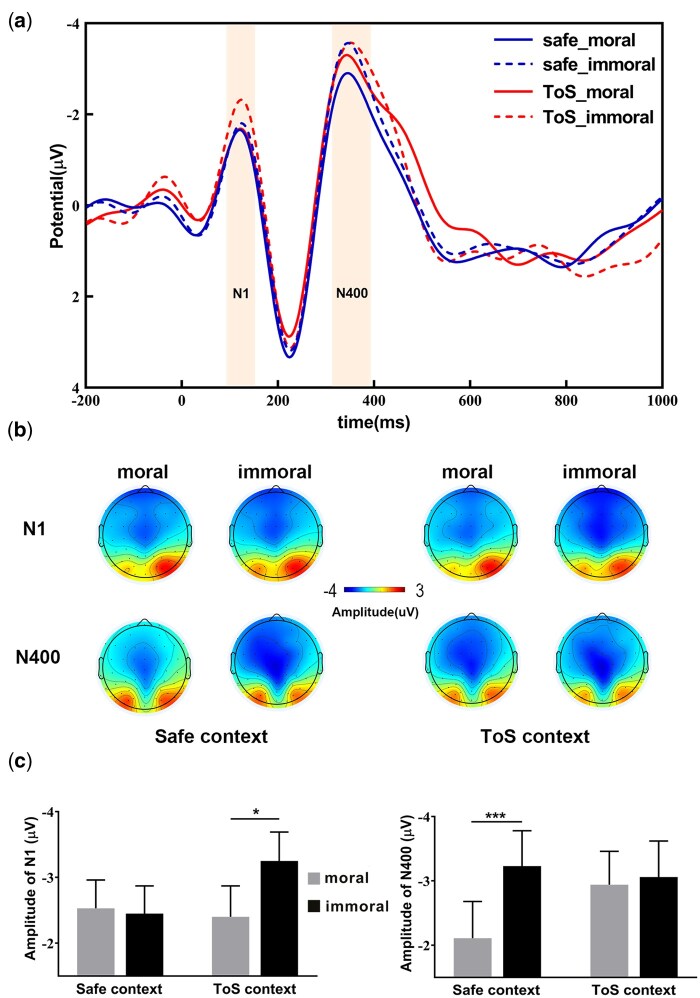
**ERP results for N1 and N400**. (a) Grand-average waveforms recorded at the Cz electrode site for the four experimental conditions; (b) Scalp topographies for the N1 (120–140 ms) and N400 (320–370 ms) components in the two contexts; (c) Amplitudes of the N1 and N400 components across the two contexts. Error bars represent the standard error. Notes: *p < 0.05, ***p < 0.001 (Bonferroni corrected).

The main effect of context was not significant (*F*  _(1,35)_ = 1.65, *P* = .21, *η_p_*^2^ = 0.05), nor was the main effect of behaviour (*F*  _(1,35)_ = 2.70, *P* = .11, *η_p_*^2^ = 0.07). However, a significant interaction between context and behaviour was observed (*F*  _(1,35)_ = 4.93, *P* = .03, *η_p_*^2^ = 0.12). Pairwise comparisons revealed that, only in the TOS context, immoral behaviour elicited significantly more negative amplitudes than moral behaviour (−2.40 ± 0.47 μV vs. −3.25 ± 0.44 μV, *P* = .014). In contrast, under the Safe context, the difference between moral and immoral behaviours was not significant (−2.53 ± 0.43 μV vs. −2.45 ± 0.42 μV, *P* = .781; Fig. 3). Notably, when *Order* was included as a covariate, the interaction between context and behaviour was attenuated and no longer significant (*F*  _(1,35)_ = 2.63, *P* = .114, *η_p_*^2^ = 0.07) was affected (see in Appendix S2).

#### P2

The main effect of context (*F*  _(1,35)_ = 0.908, *P* = .347, *η_p_*^2^ = 0.025), the main effect of behaviour (*F*  _(1,35)_ = 0.011, *P* = .917, *η_p_*^2^< 0.001) and the interaction (*F*_(1,35)_ = 0.742, *P* = .395, *η_p_*^2^ = 0.021) were not significant.

#### N400

The main effect of context was not significant (*F*  _(1,35)_ = 1.040, *P* = .315, *η_p_*^2^ = 0.029). The main effect of behaviour was significant (*F*  _(1,35)_ = 6.155, *P* = .018, *η_p_*^2^ = 0.150). The interaction was significant (*F*  _(1,35)_ = 5.694, *P* = .023, *η_p_*^2^ = 0.140). Pairwise comparisons showed that in the Safe context, immoral behaviour triggered significantly larger N400 amplitudes compared to moral behaviour (−3.23 ± 0.55 μV vs. −2.11 ± 0.57 μV, *P* < .001). Conversely, in the TOS context, there was no significant difference between moral and immoral behaviours (−2.94 ± 0.52 μV vs. −3.06 ± 0.56 μV, *P* = .75; Fig. 3). Importantly, when *Order* was included as a covariate, the interaction effect remained robust (*F*  _(1,35)_ = 7.31, *P* = .011, *η_p_*^2^ = 0.18) was still robust (see in Appendix S2).

#### P600

The main effect of context was not significant (*F*  _(1,35)_ = 3.978, *P* = .05, *η_p_*^2^ = 0.10), such that the TOS context elicited more positive amplitude than the safe context did (1.62 ± 0.31 μV vs. 1.16 ± 0.27 μV). The main effect of behaviour was not significant (*F*  _(1,35)_ = 0.982, *P* = .33, *η_p_*^2^ = 0.03). The interaction was not significant (*F*  _(1,35)_ = 0.010, *P* = .923, *η_p_*^2^< 0.001).

### Brain-behaviour relationship

Since significant interactions were observed for the N1 and N400 components, a moderation analysis was conducted to examine whether electrodermal activity (NSSCR, as a measure of anxiety) moderated the effect of context manipulation (Safe vs. TOS) on the amplitude differences of N1 and N400 evoked by moral versus immoral behaviours.

The analysis revealed that for the N1 component, the model was not significant (*F*  _(3,68)_ = 1.340, *R*^2^ = 0.056, *P* = .269). Importantly, for the N400 component, the model was significant (*F*  _(3,68)_ = 3.086, *R*^2^ = 0.12, *P* = .033). The regression equation was:


Y=0.315—0.551X+0.001Mo+0.053(X×Mo)

where *X* × Mo represents the interaction term. The main effect of the independent variable (context) was not significant, *β* = −0.551, *t*_(68)_ = −1.075, *P* = .286, and the main effect of the moderator (NSSCR) was not significant. However, the interaction term (*X × Mo*) was significant [*β*  =  0.053, *t* = 2.512, *P* = .014, 95% CI = (0.011, 0.094)], indicating that NSSCR significantly moderated the relationship between context and N400 amplitude differences.

## Discussion

In this study, we investigated individuals’ neural responses to morally salient behaviours under Safe and TOS conditions. The threat-of-shock procedure effectively induced anxiety, as demonstrated by significantly elevated subjective anxiety ratings and increased NSSCRs in the TOS condition. The robust correlation between subjective anxiety reports and objective NSSCR measures confirms the efficacy and validity of our anxiety induction paradigm.

Regarding moral perception, participants in the TOS context rated immoral behaviours as significantly less unpleasant compared to those in the Safe context, while ratings for moral behaviours remained stable across contexts. This pattern reveals a pronounced reduction in sensitivity to moral violations under anxiety, suggesting that anxious states may bias moral evaluations by attenuating the perceived severity of immoral acts. The lack of change in moral behaviour ratings further indicates that anxiety selectively affects the appraisal of immorality rather than moral practices in general, underscoring the nuanced influence of emotional states on moral cognition.

ERP findings provided critical insights into these behavioural effects. The N1 component exhibited significantly greater negative amplitudes in response to immoral versus moral behaviours within the TOS context, but not in the Safe context. Given that enhanced N1 amplitudes are indicative of heightened early attentional allocation to sensory stimuli (Hamilton et al 2014), this finding aligns with prior research demonstrating that anxiety sharpens attentional focus on threatening or salient stimuli (Nelson et al 2015). From an evolutionary standpoint, anxiety likely serves an adaptive function by facilitating rapid detection and processing of socially threatening information—such as moral violations—that may jeopardize individual rights or social standing (Prinz 2006, McEwen 2012, Crum et al 2017). Thus, the amplified N1 response in the TOS context reflects automatic, preconscious attention orienting towards socially diagnostic cues (Peng et al 2020). Notably, this interaction effect diminished after controlling for the order effect, potentially due to limited sample size or the covariate absorbing variance between context and behaviour, highlighting the need for replication with larger cohorts to confirm these neural dynamics.

In contrast, the N400 component demonstrated an inverse pattern. Under the Safe context, immoral behaviours elicited significantly larger N400 amplitudes than moral behaviours, consistent with greater semantic processing demands. This difference was absent in the TOS context, paralleling behavioural findings of reduced moral sensitivity under anxiety. The N400 is widely recognized as a neural marker of semantic integration and meaning retrieval (Chwilla et al 1995). Our result suggests that anxiety impairs the cognitive resources necessary for extracting and integrating the semantic content of immoral behaviours. According to Kutas and Federmeier (2011), the N400 amplitude reflects the cognitive effort required for meaning construction and semantic retrieval. Under the safe context, the larger N400 amplitude for immoral behaviours compared to moral ones indicates that more cognitive resources were needed to retrieve and integrate the meaning of immoral actions. However, anxiety can impair cognitive functions by diverting attentional resources and increasing the difficulty of processing information (Eysenck et al 2007). This heightened cognitive load may disrupt the semantic processing required for nuanced moral judgements, which often rely on contextual information and the ability to weigh competing ethical considerations (Greene et al. 2001). Consequently, in anxiety-inducing contexts, retrieving and integrating moral behaviours from semantic information becomes more challenging (Chaisson 2021), resulting in reduced sensitivity to moral violations during the stage of semantic processing. Notably, the robustness of the N400 effects after controlling for order effect further supports the reliability and validity of these findings, underscoring the pivotal role of anxiety in modulating semantic processing during moral evaluation.

Our behaviour-neural analysis revealed that electrodermal activity significantly modulated N400 neural responses across different contexts. Higher levels of NSSCRs reflect increased affective arousal, such as anxiety or stress (Benedek and Kaernbach 2010, Boucsein et al 2012). The moderation analysis further indicated that context manipulation—specifically, inducing anxiety through the threat of shocks—impacted the semantic processing of moral violations, as reflected in the N400 component. This finding emphasizes the integral role of physiological arousal in shaping cognitive mechanisms underlying moral judgement, illustrating the complex interplay between emotional states and semantic processing in moral cognition.

Regarding other ERP components, neither the P2 nor the P600 showed significant modulation by moral content or anxiety. The P2 is typically linked to rapid perceptual processing, while the P600 is associated with higher-order cognitive effort and reanalysis during moral judgement (Regel et al 2014, Cui et al 2018, Delogu et al 2019, Lu et al 2019). The absence of these effects may reflect a ceiling effect in cognitive processing under anxiety, where heightened arousal restricts the capacity for differentiated moral reasoning. Consequently, participants may exhibit a homogenized neural response pattern regardless of the moral valence of stimuli, suggesting that anxiety constrains late-stage evaluative processes.

This study has several limitations. First, our study focused on experimentally induced state anxiety within a controlled laboratory setting, leaving open questions regarding the generalizability of findings to individuals with trait anxiety or the interaction between trait and state anxiety. Second, despite employing a within-subject design and counterbalancing to mitigate individual differences and order effects, potential learning or fatigue effects cannot be entirely ruled out, as participants may have adjusted their moral judgement strategies upon detecting condition differences. Third, a key limitation is the absence of a non-moral negative control condition (e.g. physically aversive but morally neutral stimuli), which precludes definitive conclusions about moral specificity. Future research should directly compare anxiety’s effects on moral versus matched non-moral negative stimuli.

In sum, our findings demonstrate that anxiety significantly disrupts moral judgement, primarily by diminishing sensitivity to immoral behaviour under high-arousal states. While anxiety commonly amplifies negativity bias for general emotional stimuli (e.g. threatening faces, aversive scenes) (Baker et al 2020, Norris 2021), our results reveal a divergent pattern in moral contexts. Threat-of-shock reduced perceived unpleasantness of immoral acts while enhancing early neural vigilance, suggesting that moral judgements involve domain-specific regulatory mechanisms rather than generalized negativity amplification. Using ERP measures, we delineated two distinct processing stages in moral judgement: an early phase marked by attentional orienting and heightened vigilance to threatening stimuli (e.g. moral violations), followed by a later semantic processing phase impaired by anxiety-related depletion of cognitive resources necessary for effective integration. This impairment compromises the retrieval and synthesis of moral information, resulting in attenuated moral sensitivity.

These findings underscore anxiety’s critical role as a contextual modulator of moral evaluations, highlighting the dynamic and reciprocal relationship between emotional states and moral cognition. By elucidating how anxiety shapes the flexibility and adaptability of moral judgements, our research advances understanding of the profound influence of emotional states on individual psychology and behaviour, with implications for real-world social decision-making under stress.

## Supplementary Material

nsaf093_Supplementary_Data

## Data Availability

The data supporting the findings of this study are available at: https://doi.org/10.57760/sciencedb.25769

## References

[nsaf093-B1] Arnsten AFT , Raskind MA, Taylor FB et al The effects of stress exposure on prefrontal cortex: translating basic research into successful treatments for post-traumatic stress disorder. Neurobiol Stress 2015;1:89–99. 10.1016/j.ynstr.2014.10.002 25436222 PMC4244027

[nsaf093-B2] Bago B , De Neys W. The intuitive greater good: testing the corrective dual process model of moral cognition. J Exp Psychol Gen 2019;148:1782–801. 10.1037/xge0000533 30550336

[nsaf093-B3] Baker C , Pawling R, Fairclough S. Assessment of threat and negativity bias in virtual reality. Sci Rep 2020;10:17338. 33060767 10.1038/s41598-020-74421-1 PMC7566621

[nsaf093-B4] Balderston NL , Hale E, Hsiung A et al Threat of shock increases excitability and connectivity of the intraparietal sulcus. Elife 2017;6:e23608. 10.7554/eLife.23608 PMC5478270 28555565

[nsaf093-B5] Bartholow BD , Fabiani M, Gratton G et al A psychophysiological examination of cognitive processing of and affective responses to social expectancy violations. Psychol Sci 2001;12:197–204. 10.1111/1467-9280.00336. 11437301

[nsaf093-B6] Benedek M , Kaernbach C. A continuous measure of phasic electrodermal activity. J Neurosci Methods 2010;190:80–91. 10.1016/j.jneumeth.2010.04.028 20451556 PMC2892750

[nsaf093-B7] Berkum JJAv , Hagoort P, Brown CM. Semantic integration in sentences and discourse: evidence from the N400. J Cogn Neurosci 1999;11:657–71. 10.1162/089892999563724. 10601747

[nsaf093-B8] Białek M , De Neys W. Dual processes and moral conflict: evidence for deontological reasoners’ intuitive utilitarian sensitivity. Judgm Decis Mak 2017;12:148–67. 10.1017/S1930297500005696

[nsaf093-B9] Bolton S , Robinson OJ. The impact of threat of shock-induced anxiety on memory encoding and retrieval. Learn Mem 2017;24:532–42. 10.1101/lm.045187.117 28916628 PMC5602344

[nsaf093-B10] Boucsein W , Fowles DC, Grimnes S, et al Publication recommendations for electrodermal measurements. Psychophysiology 2012;49:1017–34. 10.1111/j.1469-8986.2012.01384.x 22680988

[nsaf093-B11] Braithwaite JJ , Watson DG, Jones R, Rowe M. A guide for analysing electrodermal activity (EDA) & skin conductance responses (SCRs) for psychological experiments. Technical report. 2013. https://www.birmingham.ac.uk/documents/college-les/psych/saal/guide-electrodermal-activity.pdf

[nsaf093-B12] Brown H , Proulx MJ, Stanton Fraser D. Hunger bias or gut instinct? Responses to judgments of harm depending on visceral state versus intuitive decision-making. Front Psychol 2020;11:2261. 10.3389/fpsyg.2020.02261 33041900 PMC7530233

[nsaf093-B13] Chaisson FM. *Neurocognitive* interactions between anticipatory anxiety and memory encoding. Master’s Theses, Louisiana State University and Agricultural & Mechanical College, Baton Rouge, 2021. https://digitalcommons.lsu.edu/gradschool_theses/5263

[nsaf093-B14] Cheung S-K. Reliability and factor structure of the Chinese version of the Depression Self-Rating Scale. Educ Psychol Meas 1996;56:142–54. 10.1177/0013164496056001011.

[nsaf093-B15] Chwilla DJ , Brown CM, Hagoort P. The N400 as a function of the level of processing. Psychophysiology 1995;32:274–85. 10.1111/j.1469-8986.1995.tb02956.x 7784536

[nsaf093-B16] Clark VP , Hillyard SA. Spatial selective attention affects early extrastriate but not striate components of the visual evoked potential. J Cogn Neurosci 1996;8:387–402. 10.1162/jocn.1996.8.5.387 23961943

[nsaf093-B17] Crum AJ , Akinola M, Martin A et al The role of stress mindset in shaping cognitive, emotional, and physiological responses to challenging and threatening stress. Anxiety Stress Coping 2017;30:379–95. 10.1080/10615806.2016.1275585 28120622

[nsaf093-B18] Cui F , Wu S, Wu H et al Altruistic and self-serving goals modulate behavioral and neural responses in deception. Soc Cogn Affect Neurosci 2018;13:63–71. 10.1093/scan/nsx138 29149322 PMC5793826

[nsaf093-B19] De Pascalis V , Arwari B, D'Antuono L, Cacace I. Impulsivity and semantic/emotional processing: an examination of the N400 wave. Clin Neurophysiol 2009;120:85–92. 10.1016/j.clinph.2008.10.008 19026592

[nsaf093-B20] Delogu F , Brouwer H, Crocker MW. Event-related potentials index lexical retrieval (N400) and integration (P600) during language comprehension. Brain Cogn 2019;135:103569. 10.1016/j.bandc.2019.05.007 31202158

[nsaf093-B21] Delorme A , Makeig S. EEGLAB: an open source toolbox for analysis of single-trial EEG dynamics including independent component analysis. J Neurosci Methods 2004;134:9–21. 10.1016/j.jneumeth.2003.10.009. 15102499

[nsaf093-B22] Ding J , Wang L, Yang Y. The dynamic influence of emotional words on sentence comprehension: an ERP study. Cogn Affect Behav Neurosci 2016;16:433–46. 10.3758/s13415-016-0403-x 26833049

[nsaf093-B23] Ellemers N , Spears R, Doosje B. Self and social identity. Annu Rev Psychol 2002;53:161–86. 10.1146/annurev.psych.53.100901.135228 11752483

[nsaf093-B24] Emerson SN , Conway CM, Özçalışkan Ş. Semantic P600—but not N400—effects index crosslinguistic variability in speakers’ expectancies for expression of motion. Neuropsychologia 2020;149:107638. 10.1016/j.neuropsychologia.2020.107638 33007360

[nsaf093-B25] Eysenck MW , Derakshan N, Santos R et al Anxiety and cognitive performance: attentional control theory. Emotion 2007;7:336–53. 10.1037/1528-3542.7.2.336 17516812

[nsaf093-B26] Gan T , Lu X, Li W et al Temporal dynamics of the integration of intention and outcome in harmful and helpful moral judgment. Front Psychol 2016;6:2022. 10.3389/fpsyg.2015.02022 26793144 PMC4708004

[nsaf093-B27] Greene J. From neural ‘is’ to moral ‘ought’: what are the moral implications of neuroscientific moral psychology? Nat Rev Neurosci 2003;4:846–9. 10.1038/nrn1224 14523384

[nsaf093-B28] Greene JD. The cognitive neuroscience of moral judgment and decision making. In: Poeppel D, Mangun GR, Gazzaniga MS (ed.), The Cognitive Neurosciences, 5th edn. MIT Press, Cambridge, MA, 2014, 1013–23.

[nsaf093-B8826392] Greene JD , Sommerville RB, Nystrom LE et al An fmri investigation of emotional engagement in moral judgment. Science 2001;293:2105–8. 10.1126/science.1062872 11557895

[nsaf093-B29] Greene JD , Morelli SA, Lowenberg K et al Cognitive load selectively interferes with utilitarian moral judgment. Cognition 2008;107:1144–54. 10.1016/j.cognition.2007.11.004 18158145 PMC2429958

[nsaf093-B30] Greene JD , Nystrom LE, Engell AD et al The neural bases of cognitive conflict and control in moral judgment. Neuron 2004;44:389–400. 10.1016/j.neuron.2004.09.027 15473975

[nsaf093-B31] Haidt J. The emotional dog and its rational tail: a social intuitionist approach to moral judgment. Psychol Rev 2001;108:814–34. 10.1037/0033-295x.108.4.814 11699120

[nsaf093-B32] Hamilton RK , Baskin-Sommers AR, Newman JP. Relation of frontal N100 to psychopathy-related differences in selective attention. Biol Psychol 2014;103:107–16. 10.1016/j.biopsycho.2014.08.012 25179538 PMC4407830

[nsaf093-B33] Hayes AF. PROCESS: a versatile computational tool for observed variable mediation, moderation, and conditional processmodeling. [white paper]. 2012. Retrieved November 22, 2024. http://www.afhayes.com/public/process2012.pdf

[nsaf093-B34] Hillyard SA , Anllo-Vento L. Event-related brain potentials in the study of visual selective attention. Proc Natl Acad Sci U S A 1998;95:781–7. 10.1073/pnas.95.3.781 9448241 PMC33798

[nsaf093-B35] Horberg EJ , Oveis C, Keltner D. Emotions as moral amplifiers: an appraisal tendency approach to the influences of distinct emotions upon moral judgment. Emotion Rev 2011;3:237–44. 10.1177/1754073911402384

[nsaf093-B36] Jeong HJ , Cho YS. Cognitive control under high threat: the effect of shock on the congruency sequence effect. Motiv Emot 2019;43:906–16. 10.1007/s11031-019-09793-7

[nsaf093-B37] Katrin S , Polzer C, Wolf OT et al Does stress alter everyday moral decision-making? Psychoneuroendocrinology 2011;36:210–9. 10.1016/j.psyneuen.2010.07.010 20692104

[nsaf093-B38] Kouchaki M , Desai SD. Anxious, threatened, and also unethical: how anxiety makes individuals feel threatened and commit unethical acts. J Appl Psychol 2015;100:360–75. 10.1037/a0037796 25243997

[nsaf093-B39] Kutas M , Federmeier KD. Thirty years and counting: finding meaning in the N400 component of the event-related brain potential (ERP). Annu Rev Psychol 2011;62:621–47. 10.1146/annurev.psych.093008.131123 20809790 PMC4052444

[nsaf093-B40] Lau EF , Phillips C, Poeppel D. A cortical network for semantics: (de)constructing the N400. Nat Rev Neurosci 2008;9:920–33. 10.1038/nrn2532 19020511

[nsaf093-B41] Li Z , Gao L, Zhao X et al Deconfounding the effects of acute stress on abstract moral dilemma judgment. Curr Psychol 2021;40:5005–18. 10.1007/s12144-019-00453-0

[nsaf093-B42] Lu J , Peng X, Liao C et al The stereotype of professional roles influences neural responses to moral transgressions: ERP evidence. Biol Psychol 2019;145:55–61. 10.1016/j.biopsycho.2019.04.007 31005611

[nsaf093-B43] Luck SJ , Gaspelin N. How to get statistically significant effects in any ERP experiment (and why you shouldn’t). Psychophysiology 2017;54:146–57. 10.1111/psyp.12639 28000253 PMC5178877

[nsaf093-B44] Luck SJ , Hillyard SA. Electrophysiological correlates of feature analysis during visual search. Psychophysiology 1994;31:291–308. 10.1111/j.1469-8986.1994.tb02218.x 8008793

[nsaf093-B45] Luo Y-J , Hu S, Weng X-C et al Effects of semantic discrimination of Chinese words on N400 component of event-related potentials. Percept Mot Skills 1999;89:185–93. 10.2466/PMS.89.5.185-193 10544413

[nsaf093-B46] Makeig S , Debener S, Onton J et al Mining event-related brain dynamics. Trends Cogn Sci 2004;8:204–10. 10.1016/j.tics.2004.03.008 15120678

[nsaf093-B47] Malle BF. Moral judgments. Annu Rev Psychol 2021;72:293–318. 10.1146/annurev-psych-072220-104358 32886588

[nsaf093-B48] McEwen BS. Brain on stress: how the social environment gets under the skin. Proc Natl Acad Sci USA 2012;109 Suppl 2:17180–5. 10.1073/pnas.1121254109 23045648 PMC3477378

[nsaf093-B49] Mende-Siedlecki P , Baron SG, Todorov A. Diagnostic value underlies asymmetric updating of impressions in the morality and ability domains. J Neurosci 2013;33:19406–15. 10.1523/JNEUROSCI.2334-13.2013 24336707 PMC6618766

[nsaf093-B50] Molinaro N , Conrad M, Barber HA et al On the functional nature of the N400: contrasting effects related to visual word recognition and contextual semantic integration. Cognitive Neuroscience 2010;1:1–7. 10.1080/17588920903373952 24168241

[nsaf093-B51] Nelson BD , Hajcak G, Shankman SA. Event-related potentials to acoustic startle probes during the anticipation of predictable and unpredictable threat. Psychophysiology 2015;52:887–94. 10.1111/psyp.12418 25703182

[nsaf093-B52] Norris CJ. The negativity bias, revisited: evidence from neuroscience measures and an individual differences approach. Soc Neurosci 2021;16:68–82. 31750790 10.1080/17470919.2019.1696225

[nsaf093-B53] Onton J , Delorme A, Makeig S. Frontal midline EEG dynamics during working memory. Neuroimage 2005;27:341–56. 10.1016/j.neuroimage.2005.04.014 15927487

[nsaf093-B54] Patil I , Larsen EM, Kichic R et al Moral cognition about harm in anxiety disorders: the importance of experienced emotion. Psychol Rep 2021;124:2501–23. 10.1177/0033294120964134 33028157

[nsaf093-B55] Paxton JM , Ungar L, Greene JD. Reflection and reasoning in moral judgment. Cogn Sci 2012;36:163–77. 10.1111/j.1551-6709.2011.01210.x. 22049931

[nsaf093-B56] Peng X , Lu J, Li L et al Three stages of perceiving consecutively moral behaviors: neurophysiological effect of agent and valence on the moral judgments. Soc Neurosci 2020;15:458–69. 10.1080/17470919.2020.1759682 32320332

[nsaf093-B57] Phelps EA , Lempert KM, Sokol-Hessner P. Emotion and decision making: multiple modulatory neural circuits. Annu Rev Neurosci 2014;37:263–87. 10.1146/annurev-neuro-071013-014119 24905597

[nsaf093-B58] Prinz J. The emotional basis of moral judgments. Philosophical Explorations 2006;9:29–43. 10.1080/13869790500492466

[nsaf093-B59] Raio CM , Hartley CA, Orederu TA et al Stress attenuates the flexible updating of aversive value. Proc Natl Acad Sci U S A 2017;114:11241–6. 10.1073/pnas.1702565114 28973957 PMC5651737

[nsaf093-B60] Regel S , Meyer L, Gunter TC. Distinguishing neurocognitive processes reflected by P600 effects: evidence from ERPs and neural oscillations. PLoS One 2014;9:e96840. 10.1371/journal.pone.0096840 24844290 PMC4028180

[nsaf093-B61] Robinson OJ , Vytal K, Cornwell BR et al The impact of anxiety upon cognition: perspectives from human threat of shock studies. Front Hum Neurosci 2013;7:203. 10.3389/fnhum.2013.00203 23730279 PMC3656338

[nsaf093-B62] Sarlo M , Lotto L, Manfrinati A et al Temporal dynamics of cognitive–emotional interplay in moral decision-making. J Cogn Neurosci 2012;24:1018–29. 10.1162/jocn_a_00146 21981668

[nsaf093-B63] Schmitz A , Grillon C. Assessing fear and anxiety in humans using the threat of predictable and unpredictable aversive events (the NPU-threat test). Nature Protocols 2012;7:527–32. 10.1038/nprot.2012.001 22362158 PMC3446242

[nsaf093-B64] Shackman AJ , Maxwell JS, McMenamin BW et al Stress potentiates early and attenuates late stages of visual processing. J Neurosci 2011;31:1156–61. 10.1523/JNEUROSCI.3384-10.2011 21248140 PMC3037336

[nsaf093-B65] Shek DTL. Reliability and factorial structure of the Chinese version of the state-trait anxiety inventory. J Psychopathol Behav Assess 1988;10:303–17. 10.1007/BF00960624

[nsaf093-B66] Shin LM , Liberzon I. The neurocircuitry of fear, stress, and anxiety disorders. Neuropsychopharmacology 2010;35:169–91. 10.1038/npp.2009.83 19625997 PMC3055419

[nsaf093-B67] Singer N , Sommer M, Wust S et al Effects of gender and personality on everyday moral decision-making after acute stress exposure. Psychoneuroendocrinology 2021;124:105084. 10.1016/j.psyneuen.2020.105084 33387970

[nsaf093-B68] Skitka LJ , Hanson BE, Morgan GS et al The psychology of moral conviction. Annu Rev Psychol 2021;72:347–66. 10.1146/annurev-psych-063020-030612 32886586

[nsaf093-B69] Starcke K , Ludwig A-C, Brand M. Anticipatory stress interferes with utilitarian moral judgment. Judgm Decis Mak 2012;7:61–8. 10.1017/S1930297500001832

[nsaf093-B70] Tinghog G , Andersson D, Bonn C et al Intuition and moral decision-making—the effect of time pressure and cognitive load on moral judgment and altruistic behavior. PLoS One 2016;11:e0164012. 10.1371/journal.pone.0164012 27783704 PMC5082681

[nsaf093-B71] Tyler TR , Blader SL. The group engagement model: procedural justice, social identity, and cooperative behavior. Pers Soc Psychol Rev 2003;7:349–61. 10.1207/S15327957PSPR0704_07 14633471

[nsaf093-B72] van Marle HJ , Hermans EJ, Qin S et al From specificity to sensitivity: how acute stress affects amygdala processing of biologically salient stimuli. Biol Psychiatry 2009;66:649–55. 10.1016/j.biopsych.2009.05.014 19596123

[nsaf093-B73] Weymar M , Bradley MM, Hamm AO et al When fear forms memories: threat of shock and brain potentials during encoding and recognition. Cortex 2013;49:819–26. 10.1016/j.cortex.2012.02.012 22483973 PMC3632083

[nsaf093-B74] White KR , Crites Jr. Jr, Taylor JH et al Wait, what? Assessing stereotype incongruities using the N400 ERP component. Social Cognitive and Affective Neuroscience 2009;4:191–8. 10.1093/scan/nsp004 19270040 PMC2686231

[nsaf093-B75] Xiaozhe P , Jiao C, Cui F et al The time course of indirect moral judgment in gossip processing modulated by different agents. Psychophysiology 2017;54:1459–71. 10.1111/psyp.12893 28543218

[nsaf093-B76] Yang S , Zhang M, Xu J et al The electrophysiology correlation of the cognitive bias in anxiety under uncertainty. Sci Rep 2020;10:11354. 10.1038/s41598-020-68427-y 32647252 PMC7347926

[nsaf093-B77] Yoder KJ , Decety J. Spatiotemporal neural dynamics of moral judgment: a high-density ERP study. Neuropsychologia 2014;60:39–45. 10.1016/j.neuropsychologia.2014.05.022 24905282 PMC4104265

[nsaf093-B78] Youssef FF , Dookeeram K, Basdeo V et al Stress alters personal moral decision making. Psychoneuroendocrinology 2012;37:491–8. 10.1016/j.psyneuen.2011.07.017 21899956

[nsaf093-B79] Zhang Z. The impact of stress on moral judgement. In: *Addressing Global Challenges-Exploring Socio-Cultural Dynamics and Sustainable Solutions in a Changing World*, 2025, 915-919. London, UK: Taylor & Francis Group. 10.1201/9781032676043-128

[nsaf093-B80] Zhao J , Harris M, Vigo R. Anxiety and intertemporal decision making: the effect of the behavioral inhibition system and the moderation effects of trait anxiety on both state anxiety and socioeconomic status. Pers Individ Dif 2016;95:29–33. 10.1016/j.paid.2016.02.024

