# Supplementary material for: Moral judgement under induced anxiety: threat-of-shock reduces sensitivity to immoral acts and alters neural processing
Source: Soc Cogn Affect Neurosci. 2025 Sep 10;20(1):nsaf093. doi: 10.1093/scan/nsaf093 (PMC12532311; doi:10.1093/scan/nsaf093)
Supplement: nsaf093_Supplementary_Data [file nsaf093_supplementary_data.docx]

| **Appendix S1**  Table 1 Sentences used in the experiment and its English translations | | | | | | |
| --- | --- | --- | --- | --- | --- | --- |
| **No.** | **Stimulus in Chinese** | **Stimulus in English** | **Classification** | **Valence** | **Pleasantness** | **Relevance** |
| 1 | 某某对老人不敬 | Someone disrespected the elderly | 1 | 2.0968 | 1.5484 | 7.5806 |
| 2 | 某某对老人照料 | Someone cared for the elderly | 2 | 7.5806 | 6.8710 | 7.3226 |
| 3 | 某某对老人鄙视 | Someone despised the elderly | 1 | 1.8710 | 1.3548 | 7.7419 |
| 4 | 某某对老人关照 | Someone attended to the elderly | 2 | 7.8065 | 6.9677 | 7.4839 |
| 5 | 某某对环保排斥 | Someone opposed environmental protection | 1 | 2.4516 | 2.0645 | 6.7419 |
| 6 | 某某对环保支持 | Someone supported environmental protection | 2 | 7.6452 | 6.5484 | 6.9677 |
| 7 | 某某路遇伤者不理睬 | Someone ignored the injured on the road | 1 | 2.8387 | 1.9032 | 6.8065 |
| 8 | 某某路遇伤者忙救助 | Someone helped the injured on the road | 2 | 8.0645 | 7.5484 | 7.8065 |
| 9 | 某某捡钱不还 | Someone kept the found money | 1 | 2.3548 | 1.9677 | 7.7742 |
| 10 | 某某捡钱归还 | Someone returned the found money | 2 | 8.0968 | 7.0968 | 8.1290 |
| 11 | 某某对国家统一反对 | Someone opposed national unity | 1 | 1.2581 | 1.3226 | 7.3226 |
| 12 | 某某对国家统一拥护 | Someone supported national unity | 2 | 8.5806 | 8.1290 | 7.3548 |
| 13 | 某某对民工歧视 | Someone despised migrant workers | 1 | 1.9032 | 1.6774 | 8.0323 |
| 14 | 某某对民工尊重 | Someone respected migrant workers | 2 | 7.7097 | 7.0645 | 7.9677 |
| 15 | 某某对弱小欺负 | Someone tormented the vulnerable | 1 | 1.7742 | 1.4516 | 8.1290 |
| 16 | 某某对弱小保护 | Someone protected the vulnerable | 2 | 7.9032 | 7.1935 | 7.7419 |
| 17 | 某某在游泳池小便 | Someone urinated in the swimming pool | 1 | 1.6452 | 1.5161 | 8.2903 |
| 18 | 某某在游泳池救人 | Someone saved lives in the swimming pool | 2 | 8.3871 | 7.9677 | 8.0323 |
| 19 | 某某在地铁上脱鞋 | Someone took off his shoes on the subway | 1 | 2.1935 | 1.4516 | 8.1290 |
| 20 | 某某在地铁上抓小偷 | Someone caught thieves on the subway | 2 | 8.1290 | 7.7097 | 7.6452 |
| 21 | 某某拿南京大屠杀开玩笑 | Someone mocked the Nanjing Massacre | 1 | 1.0645 | 1.0323 | 8.6452 |
| 22 | 某某对南京大屠杀敬畏 | Someone revered the Nanjing Massacre | 2 | 8.7419 | 7.9032 | 8.3226 |
| 23 | 某某对赌博上瘾 | Someone was addicted to gambling | 1 | 1.9355 | 1.8387 | 5.5161 |
| 24 | 某某对赌博抵制 | Someone was opposed to gambling | 2 | 7.9032 | 6.8387 | 5.8387 |
| 25 | 某某在灾区诈捐 | Someone falsely donated in disaster areas | 1 | 1.2903 | 1.1935 | 8.1290 |
| 26 | 某某在灾区捐款 | Someone donated in disaster areas | 2 | 8.3548 | 7.3226 | 7.9355 |
| 27 | 某某把他人隐私泄露 | Someone leaked others' privacy | 1 | 1.5806 | 1.3548 | 7.8710 |
| 28 | 某某对他人隐私保密 | Someone kept others' privacy | 2 | 7.8387 | 7.1613 | 7.5161 |
| 29 | 某某对朋友撒谎 | Someone lied to friends | 1 | 2.7419 | 2.0968 | 7.2903 |
| 30 | 某某对朋友仗义 | Someone stood up for friends | 2 | 7.7097 | 7.2903 | 7.3548 |
| 31 | 某某对公共财产破坏 | Someone damaged public property | 1 | 1.8065 | 1.4839 | 8.0000 |
| 32 | 某某对公共财产爱护 | Someone took care of public property | 2 | 7.5806 | 6.8387 | 7.3871 |
| 33 | 某某对别人轻蔑 | Someone treated others contemptuously | 1 | 2.0645 | 1.9355 | 7.4516 |
| 34 | 某某对别人友善 | Someone treated others kindly | 2 | 7.6452 | 7.1290 | 7.0645 |
| 35 | 某某在医院闹事 | Someone caused trouble in the hospital | 1 | 1.2903 | 1.3548 | 8.1935 |
| 36 | 某某在医院献血 | Someone donated blood in the hospital | 2 | 7.7742 | 7.0000 | 7.2903 |
| 37 | 某某对老人嘲讽 | Someone sneered at the elderly | 1 | 2.0323 | 1.5161 | 7.8065 |
| 38 | 某某为老人拎重物 | Someone carried heavy items for the elderly | 2 | 7.7419 | 6.8387 | 7.2581 |
| 39 | 某某在公交车上抢座 | Someone grabbed seats on the bus | 1 | 2.7419 | 2.0968 | 6.8710 |
| 40 | 某某在公交车上让座 | Someone offered seats on the bus | 2 | 7.9032 | 6.8710 | 7.7097 |
| 41 | 某某去外地诈骗 | Someone committed fraud in other cities | 1 | 1.5484 | 1.3871 | 7.6774 |
| 42 | 某某去外地创业 | Someone started businesses in other cities | 2 | 6.3548 | 5.2258 | 3.8387 |
| 43 | 某某用善款挥霍 | Someone squandered charity funds for personal gain | 1 | 1.3548 | 1.2903 | 8.4194 |
| 44 | 某某用善款助人 | Someone used charity funds wisely and helped many | 2 | 8.2903 | 7.4839 | 7.9355 |
| 45 | 某某对服务员吼叫 | Someone yelled at the waiter | 1 | 2.2258 | 1.6129 | 7.4194 |
| 46 | 某某对服务员微笑 | Someone smiled at the waiter | 2 | 7.3871 | 6.8710 | 7.1290 |
| 47 | 某某在比赛中喝倒彩 | Someone booed during the match | 1 | 2.6774 | 2.2581 | 6.6129 |
| 48 | 某某在比赛中鼓劲 | Someone cheered during the match | 2 | 7.3548 | 6.6774 | 6.2581 |
| 49 | 某某对交警粗鲁 | Someone was rude to traffic police | 1 | 2.1290 | 1.6452 | 7.6774 |
| 50 | 某某对交警礼貌 | Someone was polite to traffic police | 2 | 7.3548 | 6.6129 | 7.5484 |
| 51 | 某某对腐败纵容 | Someone connived corruption | 1 | 1.6774 | 1.5484 | 7.2581 |
| 52 | 某某对腐败抗议 | Someone protested corruption | 2 | 8.0968 | 7.1935 | 7.4194 |
| 53 | 某某对评委行贿 | Someone bribed the judges | 1 | 1.6129 | 1.4839 | 7.9355 |
| 54 | 某某对评委致谢 | Someone thanked the judges | 2 | 7.1613 | 6.2903 | 6.9355 |
| 55 | 某某在成年后啃老 | Someone lived off his parents after adulthood | 1 | 2.7097 | 1.9677 | 6.9677 |
| 56 | 某某在成年后自立 | Someone earned his own living after adulthood | 2 | 7.5161 | 7.0645 | 6.1290 |
| 57 | 某某在面试中造假 | Someone cheated in job interviews | 1 | 1.8710 | 1.6129 | 7.8065 |
| 58 | 某某在面试中诚实 | Someone was honest in job interviews | 2 | 7.4516 | 6.6774 | 7.4194 |
| 59 | 某某对同行诋毁 | Someone slandered his peers | 1 | 2.1290 | 1.7419 | 8.0323 |
| 60 | 某某对同行赞扬 | Someone praised his peers | 2 | 7.4194 | 6.6774 | 7.2258 |
| 61 | 某某在辩论中辱骂 | Someone insulted during debates | 1 | 2.1290 | 1.5161 | 8.0645 |
| 62 | 某某在辩论中调解 | Someone mediated during debates | 2 | 7.2581 | 6.5484 | 6.7419 |
| 63 | 某某将废旧物乱丢 | Someone littered wastes | 1 | 2.4839 | 1.9032 | 7.4194 |
| 64 | 某某将废旧物回收 | Someone recycled wastes | 2 | 7.4194 | 6.8065 | 7.2258 |
| 65 | 某某在工作中偷懒 | Someone slacked off at work | 1 | 3.1613 | 2.4516 | 6.0323 |
| 66 | 某某在工作中努力 | Someone worked hard at work | 2 | 7.2258 | 6.6129 | 5.8710 |
| 67 | 某某对老人斥责 | Someone scolded the elderly | 1 | 2.1935 | 1.8065 | 7.1935 |
| 68 | 某某对老人耐心 | Someone was patient with the elderly | 2 | 7.5806 | 6.8710 | 7.2581 |
| 69 | 某某跟邻居恶斗 | Someone fought with neighbors | 1 | 2.6129 | 2.1935 | 6.6774 |
| 70 | 某某跟邻居和睦 | Someone got along well with neighbors | 2 | 7.5161 | 6.6774 | 6.8710 |
| 71 | 某某在升旗时打电话 | Someone made phone calls during flag raising ceremony | 1 | 1.8710 | 1.6452 | 7.3226 |
| 72 | 某某在升旗时敬礼 | Someone saluted during flag raising ceremony | 2 | 8.0645 | 7.2581 | 7.2258 |
| 73 | 某某对伴侣打骂 | Someone hit and yelled at his partner | 1 | 1.6129 | 1.5161 | 7.8065 |
| 74 | 某某对伴侣贴心 | Someone was considerate to his partner | 2 | 7.9032 | 7.6129 | 7.1290 |
| 75 | 某某给贫困生白眼 | Someone looked down upon impoverished students | 1 | 1.9355 | 1.5484 | 7.8387 |
| 76 | 某某给贫困生资助 | Someone supported impoverished students | 2 | 7.8710 | 7.3871 | 7.5161 |
| 77 | 某某在图书馆喧闹 | Someone made noise in the library | 1 | 1.9032 | 1.3871 | 8.1935 |
| 78 | 某某在图书馆做义工 | Someone volunteered in the library | 2 | 7.6452 | 6.7419 | 7.3548 |
| 79 | 某某对爱人施暴 | Someone abused his lover | 2 | 8.0968 | 7.7097 | 6.9032 |
| 80 | 某某对爱人温柔 | Someone adored his lover | 1 | 1.5484 | 1.3548 | 8.2903 |
| 81 | 某某对孤寡老人冷漠 | Someone was indifferent to the elderly living alone | 1 | 2.6452 | 1.9677 | 7.2581 |
| 82 | 某某给孤寡老人读报 | Someone read newspapers to the elderly living alone | 2 | 7.3226 | 6.8065 | 7.0968 |
| 83 | 某某对新人欺压 | Someone bullied new employees | 1 | 2.2258 | 1.6129 | 7.5484 |
| 84 | 某某对新人照顾 | Someone guided new employees | 2 | 7.2258 | 7.0323 | 6.6129 |
| 85 | 某某开车时过斑马线加速 | Someone sped up when driving over a crosswalk | 1 | 1.9032 | 1.5161 | 7.1613 |
| 86 | 某某开车时过斑马线减速 | Someone slowed down when driving over a crosswalk | 2 | 7.4516 | 6.9032 | 6.4839 |
| 87 | 某某在车祸发生时围观 | Someone just gawked during a car accident | 1 | 3.1935 | 2.2581 | 6.6129 |
| 88 | 某某在车祸发生时救援 | Someone provided rescue during a car accident | 2 | 8.1290 | 7.9032 | 7.8710 |
| 89 | 某某遇到事故旁观 | Someone bystood accidents | 1 | 2.7419 | 2.2903 | 7.0645 |
| 90 | 某某遇到事故援助 | Someone assisted during accidents | 2 | 8.0323 | 7.5161 | 7.8387 |
| 91 | 某某对稀有动物捕猎 | Someone hunted rare animals | 1 | 1.8387 | 1.5806 | 7.3226 |
| 92 | 某某对稀有动物保护 | Someone protected rare animals | 2 | 7.7419 | 7.0323 | 6.9677 |
| 93 | 某某在过马路时翻栏杆 | Someone climbed over railings when crossing the street | 1 | 2.4516 | 2.1935 | 7.2258 |
| 94 | 某某在过马路时扶老人 | Someone helped the elderly when crossing the street | 2 | 7.7419 | 6.7742 | 7.7097 |
| 95 | 某某在过马路时闯红灯 | Someone ran the red light when crossing the street | 1 | 2.2258 | 1.8065 | 7.1613 |
| 96 | 某某在过马路时等红灯 | Someone waited for the red light when crossing the street | 2 | 7.2581 | 5.9355 | 6.4839 |
| 97 | 某某遇到问题逃避 | Someone evaded responsibility when facing problems | 1 | 2.5161 | 1.7742 | 6.2903 |
| 98 | 某某遇到问题负责 | Someone took responsibility when facing problems | 2 | 7.6452 | 7.0968 | 7.0323 |
| 99 | 某某对善行讥讽 | Someone mocked good deeds | 1 | 1.9355 | 1.4839 | 7.9032 |
| 100 | 某某对善行声援 | Someone supported good deeds | 2 | 7.9032 | 7.1290 | 7.3871 |
| 101 | 某某对家人发火 | Someone was mad at his family | 1 | 2.4516 | 1.7742 | 6.6129 |
| 102 | 某某对家人体贴 | Someone cared for his family | 2 | 7.7419 | 7.4194 | 6.6129 |
| 103 | 某某对恩师翻脸 | Someone turned against his mentor | 1 | 2.3548 | 2.0000 | 6.9677 |
| 104 | 某某对恩师感激 | Someone appreciated his mentor | 2 | 7.7419 | 6.7742 | 7.0968 |
| 105 | 某某在吃饭时浪费 | Someone wasted food during meals | 1 | 2.6452 | 2.1613 | 7.0000 |
| 106 | 某某在吃饭后打包 | Someone packed leftovers after meals | 2 | 6.9032 | 6.3226 | 6.3871 |
| 107 | 某某对动物砸石头 | Someone threw stones at animals | 1 | 1.6129 | 1.2903 | 7.7742 |
| 108 | 某某给动物喂食 | Someone fed animals | 2 | 6.9032 | 6.3226 | 5.8065 |
| 109 | 某某对前辈无礼 | Someone was rude to seniors | 1 | 2.0323 | 1.6129 | 7.8387 |
| 110 | 某某对前辈敬重 | Someone respected seniors | 2 | 7.9032 | 6.9032 | 7.5806 |
| 111 | 某某鼓励别人拜金 | Someone encouraged others to pursue money worship | 1 | 2.2258 | 1.8065 | 7.4194 |
| 112 | 某某鼓励别人进步 | Someone encouraged others to improve themselves | 2 | 7.6452 | 7.0645 | 7.1290 |
| 113 | 某某对爱人不忠 | Someone cheated on his spouse | 1 | 1.1935 | 1.1613 | 8.4194 |
| 114 | 某某对爱人忠心 | Someone was loyal to his spouse | 2 | 8.1290 | 7.7742 | 7.7419 |
| 115 | 某某对弱势者藐视 | Someone showed contempt for the weak | 1 | 2.2581 | 1.6774 | 7.9032 |
| 116 | 某某对弱势者同情 | Someone showed compassion for the weak | 2 | 7.4194 | 6.9032 | 7.4516 |
| 117 | 某某下楼梯时推人 | Someone pushed people when going downstairs | 1 | 1.4516 | 1.3226 | 8.3548 |
| 118 | 某某下楼梯时右行 | Someone kept to the right when going downstairs | 2 | 6.7419 | 5.9032 | 6.3226 |
| 119 | 某某在生活中虚荣 | Someone lived a vain life | 1 | 3.0968 | 2.0968 | 5.7097 |
| 120 | 某某在生活中朴实 | Someone lived a simple life | 2 | 6.9677 | 6.4194 | 5.7419 |

Note: “1” in Classification represent for moral behavior, “2” for immoral behavior.

**Appendix S2**

For the concern of order, firstly, we conducted a linear regression analysis with order as the predictor and the difference between TOS and Safe anxiety ratings (delta_anxiety) as the outcome. The results showed that order did not significantly predict the difference in anxiety ratings (t = 1.097, *β* = 0.19, *p* = 0.28), indicating that the feelings of anxiety was not significantly influence by the order.

For ERPs, we conducted a 2 (Context: TOS vs. Safe) × 2 (Behavior: Moral vs. Immoral) repeated-measures ANOVA on the N1/N400 component, with Order of context included as a covariate in the model. The results are below:

**N1:** the main effect of context was not significant (*F* _(1,35)_ = 2.97, *p* = 0.094, *η_p_^2^* = 0.08), the interaction of context × order was not significant (*F* _(1,35)_ = 1.31, *p* = 0.26, *η_p_^2^*= 0.04). The main effect of behavior was significant (*F* _(1,35)_ = 4.64, *p* = 0.038, *η_p_^2^* = 0.07), the main effect of order was not significant (*F* _(1,35)_ = 0.73, *p* = 0.398, *η_p_^2^* = 0.02) and the interaction of behavior × order was not significant (*F* _(1,35)_ = 1.88, *p* = 0.18, *η_p_^2^*= 0.05). The interaction of context × behavior was not significant (*F* _(1,35)_ = 2.63, *p* = 0.114, *η_p_^2^*= 0.07), and the interaction of context × behavior × order was not significant (*F* _(1,35)_ < 0.001, *p* = 0.989, *η_p_^2^* < 0.001).

The effect size (the interaction of context × order) was decreased, after the addition of Order (*η_p_^2^* decreased from 0.12 to 0.07). Order did not significantly change the pattern of main effect of context (*ps* > 0.1), but increased the statistical significance of the Behavior main effect (formerly *p* = 0.11 → now *p* = 0.038).

**N400:** the main effect of context was not significant (*F* _(1,35)_ = 0.72, *p* = 0.402, *η_p_^2^* = 0.02), the interaction of context × order was not significant (*F* _(1,35)_ = 0.46, *p* = 0.502, *η_p_^2^*= 0.013). The main effect of behavior was significant (*F* _(1,35)_ = 12.66, *p* = 0.001, *η_p_^2^* = 0.27), the main effect of order was not significant (*F* _(1,35)_ = 0.97, *p* = 0.33, *η_p_^2^* = 0.03) and the interaction between behavior × order was significant (*F* _(1,35)_ = 4.76, *p* = 0.036, *η_p_^2^* = 0.123). The interaction of context × behavior was significant (*F* _(1,35)_ = 7.31, *p* = 0.011, *η_p_^2^*= 0.18). The interaction of context × behavior × order was not significant (*F* _(1,35)_ = 1.87, *p* = 0.18, *η_p_^2^*= 0.052). The interaction effect remained significant, suggesting that the moderating effect of context × behavior is robust.
